# Modulating food craving-related neural oscillations using tACS: study protocol for a randomized sham-controlled trial

**DOI:** 10.3389/fnhum.2025.1612062

**Published:** 2025-07-30

**Authors:** Milos Ljubisavljevic, Fransina C. King, Fatima Yousif Ismail, Yauhen Statsenko, Shahid Bashir, Osama Abdullah, Bas Rokers

**Affiliations:** ^1^Department of Physiology, College of Medicine and Health Sciences (CMHS), United Arab Emirates University, Al Ain, United Arab Emirates; ^2^ASPIRE Precision Medicine Research Institute Abu Dhabi, United Arab Emirates University, Al Ain, United Arab Emirates; ^3^Department of Pediatrics, College of Medicine and Health Sciences (CMHS), United Arab Emirates University, Al Ain, United Arab Emirates; ^4^Department of Radiology, College of Medicine and Health Sciences (CMHS), United Arab Emirates University, Al Ain, United Arab Emirates; ^5^Neuroscience Center, King Fahad Specialist Hospital, Dammam, Saudi Arabia; ^6^Center for Brain and Health, New York University Abu Dhabi, Abu Dhabi, United Arab Emirates

**Keywords:** food craving, tACS, fMRI, DLPFC, insula, anterior cingulate cortex

## Abstract

**Introduction:**

Food addiction is characterized by heightened craving and impaired inhibitory control, contributing to compulsive eating and obesity. Existing behavioral and pharmacological interventions often fail to achieve lasting effects. Transcranial alternating current stimulation (tACS), by modulating neural oscillations and connectivity, offers a novel, non-invasive approach for regulating craving-related neural circuits.

**Objectives:**

This study aims to evaluate the effects of fixed-frequency tACS targeting the dorsal lateral prefrontal cortex (DLPFC), anterior cingulate cortex (ACC), and insula on food cravings, inhibitory control, and related neural oscillations. The trial will assess whether tACS can reduce craving intensity and enhance inhibitory performance in individuals with varying degrees of food addiction severity.

**Methods:**

We will conduct a randomized, double-blind, sham-controlled trial involving 175 participants aged 20–55 years, stratified by food addiction status (FA+ vs. FA-) using the Yale Food Addiction Scale (YFAS 2.0). Participants will receive tACS at alpha (10 Hz) or theta (6 Hz) frequency over the DLPFC, ACC, or insula for seven consecutive days. Electroencephalography (EEG) and functional magnetic resonance imaging (fMRI) will be conducted pre- and post-intervention. Primary outcomes include changes in craving intensity and inhibitory control. Secondary outcomes include alterations in neural oscillations and functional connectivity.

**Discussion:**

We hypothesize that theta-tACS over the ACC and insula will enhance inhibitory control and interoceptive awareness, while alpha-tACS over the DLPFC will improve top-down regulatory processes. This protocol aims to clarify the neural mechanisms underlying food cravings and evaluate tACS as a promising intervention for compulsive eating.

## 1 Introduction

Food addiction is characterized by the compulsive overconsumption of highly palatable foods, typically rich in sugar, fat, and salt (Gearhardt et al., 2016). Often overlooked in clinical contexts, FA is increasingly implicated in the global rise of obesity and related metabolic disease, including type 2 diabetes, cardiovascular disorders, and some cancers (Almarshad et al., 2022; Bonaccio et al., 2022; Dinu and Martini, 2023; Soroceanu et al., 2023).

Food addiction shares neurobiological and behavioral features with substance use disorders (SUD), including impaired control, withdrawal, and continued use despite adverse consequences (Gearhardt et al., 2011; Hone-Blanchet and Fecteau, 2014; Gordon et al., 2018; Lampert and Novelle, 2023). Craving intensity in FA is associated with neural mechanisms governing rewards, emotion regulation, and inhibition. Neuroimaging studies have implicated the dorsolateral prefrontal cortex (DLPFC), anterior cingulate cortex (ACC), and insula as core nodes in these craving-related circuits (Parvaz et al., 2011; Koob and Volkow, 2016). Disruption within these networks may contribute to heightened craving and impulsivity (Kim and Lee, 2010; Wiedemann et al., 2021).

Specifically, the desynchronization of theta (4–8 Hz) oscillations in the ACC and insula and alpha (8–12 Hz) oscillations in the DLPFC have been associated with substance craving and addiction behavior (Berke, 2009; Li et al., 2017; Marinkovic and Rosen, 2022; Tian et al., 2024).

Transcranial alternating current stimulation offers a novel, frequency-specific approach to modulate these oscillations. Unlike transcranial direct current stimulation (tDCS) or repetitive transcranial magnetic stimulation (rTMS), tACS can entrain endogenous rhythms and enhance synchronization across distributed neural networks (Herrmann et al., 2013; Vosskuhl et al., 2018). Recent studies suggest that tACS can increase coherence within craving-related networks, particularly in prefrontal regions (Dunlop et al., 2016).

This protocol builds on earlier work showing that neuromodulation can affect craving and self-regulation but overcomes the limitations of static stimulation paradigms by engaging dynamic, frequency-tuned neural circuits (Vosskuhl et al., 2018; Song et al., 2019; Mehta et al., 2023). Through targeting theta and alpha oscillations in functionally distinct regions, we aim to dissect the mechanistic underpinnings of craving modulation and inform the development of targeted neuromodulatory interventions for obesity and compulsive eating behavior.

The primary aim of this study is to evaluate whether tACS targeting the DLPFC, ACC, or insula can modulate craving-related oscillatory activity and enhance inhibitory control in individuals with varying degrees of food addiction. We hypothesize that theta-frequency stimulation of the ACC and insula will strengthen inhibitory control mechanisms and interoceptive awareness, while alpha-frequency stimulation of the DLPFC will enhance top-down cognitive regulation. By investigating these site- and frequency-specific effects, this study seeks to advance our mechanistic understanding of craving-related network dynamics and inform the development of non-invasive neuromodulatory interventions for compulsive eating behavior.

## 2 Methods

### 2.1 Study design

This randomized, double-blind, sham-controlled, parallel-group trial is designed to investigate the effects of fixed-frequency transcranial alternating current stimulation (tACS) on food cravings, inhibitory control, and associated neural oscillations. The study includes participants with and without food addiction, classified according to the Yale Food Addiction Scale 2.0 (YFAS 2.0).

Participants classified as food-addicted (FA+) will be randomly assigned to one of four intervention arms: (1) active tACS targeting the anterior cingulate cortex (ACC) at a theta frequency of 6 Hz, (2) active tACS targeting the dorsolateral prefrontal cortex (DLPFC) at an alpha frequency of 10 Hz, (3) active tACS targeting the insula at a theta frequency of 6 Hz, or (4) sham tACS serving as a placebo control.

Participants classified as non-addicted (FA-) will be assigned to a separate fifth group. This group will follow a within-subject, counterbalanced crossover design in which each participant receives both active and sham stimulation across two sessions. Sessions will be scheduled on consecutive days, with a 24-h interval, and the stimulation order randomized to mitigate order effects. This approach enables within-subject comparison of stimulation effects in individuals without food addiction and allows for additional between-group analyses contrasting FA+ and FA- responses. Furthermore, the crossover approach in the FA- group is selected to reduce unnecessary stimulation exposure and focus statistical power on the primary FA+ comparisons, while still enabling exploratory within-subject analyses in non-addicted participants.

This design allows for direct comparisons between active and sham conditions and across cortical targets while also enabling exploration of craving-related network dynamics in the presence and absence of FA.

Randomization will be conducted using a computerized block randomization algorithm, stratified by food addiction status (FA+ vs. FA-). To ensure allocation concealment, the randomization sequence will be generated by a researcher not involved in data collection or analysis. All participants, tACS administrators, and outcome assessors will remain blind to group allocation throughout the study.

### 2.2 Participants

#### 2.2.1 Eligibility criteria

Participants will be adults between the ages of 20 and 55 years with a Body Mass Index (BMI) of 18.5 or higher. All participants must report high levels of food cravings and demonstrate English language proficiency sufficient to complete study procedures. To ensure physiological stability, individuals must have maintained a body weight within ± 5% of their average over the past 3 months.

Food addiction status will be assessed using the YFAS 2.0. Participants with a YFAS 2.0 score ≤ 1 will be classified as non-addicted (FA-), while those scoring ≥ 2 will be classified as food-addicted (FA+), following validated cutoff recommendations (Gearhardt et al., 2016).

#### 2.2.2 Exclusion criteria

Participants will be excluded if they have any contraindications to tACS or magnetic resonance imaging (MRI), including implanted electronic devices, metal implants in the head, or a history of seizures. Individuals with current or past diagnoses of neurological or psychiatric disorders (e.g., epilepsy, major depression, bipolar disorder, schizophrenia), as well as those meeting the DSM-5 criteria for a SUD, will be excluded. The use of medications that may influence neural connectivity or craving regulation, including psychoactive agents, appetite suppressants, and hormonal therapies, will also result in exclusion.

Additional exclusion criteria include participation in structured weight-loss programs; chronic medical conditions affecting metabolism (e.g., diabetes, thyroid dysfunction, renal disease, autoimmune disorders); and pregnancy, breastfeeding, or postmenopausal status. These exclusions aim to minimize confounding variables and ensure participant safety.

#### 2.2.3 Recruitment and screening

Participants will be recruited through university mailing lists, social media platforms, and targeted community outreach efforts. Interested individuals will first complete an online prescreening survey assessing basic eligibility, including age, BMI, craving intensity, and food addiction status using the YFAS 2.0.

Those meeting the initial criteria will be invited for an in-person screening, during which eligibility will be confirmed through structured interviews and validated behavioral assessments. These will include the State Food Craving Questionnaire (S-FCQ), Trait Food Craving Questionnaire (T-FCQ), Food Craving Inventory (FCI), and the Center for Epidemiologic Studies Depression Scale – Revised (CESD-R). The CESD-R will be used to rule out significant depressive symptoms that could influence craving or intervention response.

#### 2.2.4 Ethical approval and informed consent

Written informed consent will be obtained from all participants prior to enrollment. The study will be conducted in accordance with the Declaration of Helsinki and has been submitted for approval to both the Abu Dhabi Health Research and Technology Committee (ADHRTC) and the United Arab Emirates University Human Research Ethics Committee (UAEU-HREC).

#### 2.2.5 Sample size and power considerations

The sample size was calculated based on prior studies investigating neuromodulation effects on craving-related behavior and oscillatory activity (Song et al., 2019, 2022; Althubaiti, 2022; Kim et al., 2023; Gairola et al., 2024; Arifin and Yaacob, 2025; Sánchez-Garrido Campos et al., 2025). Assuming a moderate effect size (Cohen’s f = 0.25), an alpha level of 0.05, and a desired power of 0.80, a minimum of 35 participants per group is required to detect statistically significant differences in the primary behavioral outcome.

To account for potential attrition and non-compliance, a 20% dropout rate was incorporated into the calculation, yielding a final target of 175 participants distributed across the five study groups. This includes 140 participants in the FA+ parallel groups and 35 in the FA- crossover group.

Given the multimodal nature of data collection, including repeated electroencephalography (EEG) and functional magnetic resonance imaging (fMRI) measurements, a sensitivity analysis was also conducted. This analysis confirmed that the study remains adequately powered to detect within-subject changes in neural and behavioral outcomes while maintaining sufficient statistical power for between-group comparisons.

### 2.3 Intervention

Participants will receive tACS targeting one of three cortical regions: the ACC, DLPFC, or insula. These targets were selected based on their involvement in the triadic model of craving, representing the impulsive, reflective, and interoceptive systems, respectively (Christie and Bechara, 2020).

Stimulation will be delivered at fixed frequencies supported by the literature on oscillation-specific effects of tACS. The DLPFC will be stimulated at 10 Hz (alpha frequency), while the ACC and insula will receive 6 Hz (theta frequency) stimulation. This frequency choice aligns with prior findings suggesting functional specificity of alpha oscillations in top-down control and theta oscillations in cognitive conflict and interoception (Stecher et al., 2021; Vogeti et al., 2022; Wischnewski et al., 2023).

The stimulation intensity will vary by region, reflecting anatomical depth and field modeling data. For the DLPFC and ACC, the peak-to-peak current will range between 1.5 and 2 mA. However, due to its deeper cortical location, the insula will be stimulated with higher intensities, between 8 and 9 mA peak-to-peak. This is consistent with recent human studies demonstrating the feasibility and tolerability of higher-output tACS for reaching deep brain structures (Shan et al., 2023; Wischnewski et al., 2023). All sessions will last 20 min and occur once daily over seven consecutive days.

Individualized modeling will be conducted using T1-weighted structural MRI scans to generate finite element models (FEMs). Electrode montages will then be optimized using ROAST (Realistic vOlumetric Approach to Simulate Transcranial electric stimulation) (Huang et al., 2019), allowing simulation of electric field distributions within each participant’s head. The modeling will ensure that target field strengths exceed 0.4 mV/mm, a threshold associated with effective neuromodulation (Wischnewski et al., 2023). If predicted intensities fall below this threshold in any region, montage adjustments will be implemented prior to stimulation. HD-Explore, an online visualization tool, will be used to confirm current flow patterns and facilitate replication by other researchers.

In the sham condition, electrodes will be placed according to the international 10–20 system (F3/F4). The current will ramp up over 30 s and then be reduced to zero for the remainder of the session, followed by a final 30-s ramp-down. This approach preserves blinding by mimicking the initial somatosensory sensations associated with active tACS (Ljubisavljevic et al., 2022).

All sessions will be conducted by trained personnel, and adverse events will be monitored throughout.

### 2.4 Outcome measures

**FIGURE 1:**
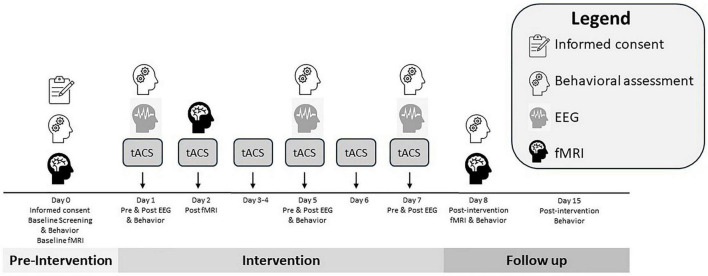
Overview of the study timeline, including screening, intervention, and follow-up phases. This schematic illustrates the flow of participant activities and data collection across all phases of the study. Screening and baseline evaluations (Day 0) include eligibility verification, informed consent, structural MRI, and administration of behavioral questionnaires (YFAS 2.0, T-FCQ, CESD-R). The intervention phase (Days 1–7) involves daily tACS, with EEG performed on Days 1, 5, and 7, and behavioral assessments (S-FCQ, FCI, Go/No-Go task) performed on Days 1, 5, and 8. An fMRI will be performed post-tACS on Day 2. Post-intervention imaging (fMRI) and follow-up behavioral measures occur on Day 8. A follow-up assessment on Day 15 evaluates potential sustained effects and adverse events.

The primary outcomes of this study are changes in food craving intensity and inhibitory control following the tACS intervention. Craving will be assessed using the State Food Craving Questionnaire (S-FCQ) and the Food Craving Inventory (FCI), both administered before and immediately after stimulation sessions on Days 1 and 5 (Figure 1). Inhibitory control will be evaluated using a computerized Go/No-Go task at the same time points. These behavioral measures will provide insight into the acute and cumulative effects of tACS on craving regulation and impulse control.

Secondary outcomes will focus on neural mechanisms underlying these behavioral changes. EEG will be recorded immediately before and after stimulation on Days 1, 5, and 7 to capture neurophysiological aftereffects (Figure 1). Key EEG parameters include frequency-specific power changes indicative of entrainment, phase-locking value (PLV), and event-related potentials (ERPs). Data will be analyzed at both sensor and source levels using established pipelines.

Functional magnetic resonance imaging will be used to assess changes in functional connectivity within craving-related neural networks. Imaging will be conducted pre- and post-intervention (Day 0 and Day 8) and will include both resting-state scans and a cue-reactivity paradigm (Figure 1). Connectivity will be examined using seed-based correlation and independent component analysis (ICA), targeting the impulsive (ACC), reflective (DLPFC), and interoceptive (insula) systems, which correspond to the stimulation targets.

Craving-related traits will be assessed using the T-FCQ and the YFAS 2.0, administered at baseline (Day 0), and again during follow-up (Day 15) (Figure 1). These measures will provide a broader understanding of individual differences in craving susceptibility and their potential modulation by tACS.

Finally, follow-up assessments on Day 15 will evaluate whether observed behavioral and neural changes persist beyond the immediate intervention phase. This longitudinal component is designed to assess the sustainability of tACS effects and explore delayed neuroplastic adaptations.

### 2.5 Data collection and analysis

Behavioral data will be collected at multiple time points to evaluate the impact of tACS on food cravings and inhibitory control. The S-FCQ, FCI, and Go/No-Go task will be administered pre- and post-stimulation on Days 1 and 5, and again on Day 8 to assess short-term changes in craving and impulse regulation (Figure 1). Broader trait-level assessments, including the YFAS 2.0 and the T-FCQ, will be administered at baseline (Day 0) and again during the follow-up session on Day 15 to examine long-term or sustained effects (Figure 1).

Electroencephalographic data will be recorded on Days 1, 5, and 7 (Figure 1) using a 64-channel dry electrode cap following the international 10–20 system. Each EEG session will include 5 min of resting-state recording with eyes open, followed by 5 min of viewing food-related and neutral images. Recordings will be conducted both before and after stimulation to detect aftereffects. EEG data will be acquired using the ANT Neuro system at a sampling rate of 1064 Hz. Impedance will be maintained below 10 kΩ. Signal preprocessing and artifact correction will be performed using standard pipelines in EEGLAB and custom MATLAB scripts. Key neural indices will include spectral power, PLV, and ERPs.

Functional MRI data will be acquired using a Siemens 3T Prisma scanner with a 64-channel head coil. Imaging sessions will occur at baseline (Day 0), immediately post-tACS on Day 2, and again post-intervention (Day 8) (Figure 1). Each session will include an 8-min resting-state scan and a cue-reactivity task involving the passive viewing of highly palatable food and neutral images. The cue task will use a block design format with craving intensity ratings collected after each block. BOLD signal changes will be analyzed using Statistical Parametric Mapping 12 (SPM12) and FMRIB Software Library (FSL), with preprocessing steps including realignment, normalization, and spatial smoothing. Functional connectivity will be assessed using seed-based correlation and ICA approaches, focusing on DLPFC, ACC, and insula as predefined regions of interest.

All adverse events or discomforts related to tACS will be monitored daily throughout the intervention phase. Any participant experiencing persistent adverse effects will be withdrawn from the study and referred for appropriate follow-up care.

Data management will be conducted in accordance with ethical data protection standards. All data will be stored on a secure, encrypted server with access limited to authorized personnel. Personal identifiers will be removed and replaced with anonymized participant codes. Missing data will be handled using multiple imputations via chained equations (MICE), assuming data are missing at random. Sensitivity analyses will be conducted to test the robustness of findings under different imputation models.

All statistical analyses will follow an intention-to-treat approach. The primary outcome–change in S-FCQ and FCI scores–will be analyzed using repeated measures ANOVA, with time (pre/post) and group (stimulation target and sham) as factors. *Post hoc* comparisons will be corrected using the Bonferroni method. Changes in Go/No-Go performance will be analyzed using ANCOVA, adjusting baseline reaction times and error rates. EEG parameters will be analyzed using linear mixed-effects models to account for within-subject correlations across sessions. Functional connectivity changes in fMRI will be assessed using seed-based and ICA models, with between-group comparisons conducted using *t*-tests or mixed models where appropriate. Longitudinal changes in YFAS and T-FCQ scores will be modeled using hierarchical linear regression.

Exploratory analyses will examine whether individual differences in sex, BMI, and baseline craving severity moderate the effects of tACS. Clustering methods will also be used to identify subgroups based on EEG and fMRI response profiles.

## 3 Discussion

This study aims to address a critical gap in neuromodulation research by investigating the efficacy of tACS in modulating food cravings and neural oscillations. While prior studies have demonstrated that non-invasive brain stimulation techniques such as tDCS and rTMS can influence craving-related behaviors, their effects on oscillatory coherence and large-scale network dynamics remain less explored (Parvaz et al., 2011; Koob and Volkow, 2016; Schwab et al., 2019). tACS, in contrast, offers the unique advantage of directly entraining neural oscillations at behaviorally relevant frequencies, potentially leading to more robust and sustained changes in food cravings.

Craving-related behaviors are increasingly understood as arising from dysregulated interactions between cortical control regions and subcortical rewards circuits. Disrupted connectivity between the prefrontal cortex and structures such as the striatum and amygdala has been implicated in impulsivity and the inability to exert inhibitory control over salient food cues (Chen et al., 2017; Burnette et al., 2019; Liu et al., 2023). By applying tACS over key cortical hubs, including the DLPFC, ACC, and insula at behaviorally relevant frequencies (alpha and theta), this study targets three components of the triadic model of craving: the reflective, impulsive, and interoceptive systems. This frequency-region pairing allows for a hypothesis-driven investigation of how specific oscillatory regimes influence discrete components of craving regulation.

Previous work has linked elevated theta and beta oscillatory activity in fronto-striatal and amygdala networks with increased craving and diminished inhibitory control (Cavanagh and Frank, 2014; Koob and Volkow, 2016; Zavala et al., 2017). tACS has already demonstrated efficacy in modulating such rhythms in cognitive domains such as attention and working memory (Herrmann et al., 2013; Wischnewski and Schutter, 2015) and may exert similar benefits in the context of food addiction. If successful, this study will provide empirical evidence that modulating the frequency-specific dynamics of craving-related networks can lead to measurable behavioral and neural changes in food-related impulse regulation.

By combining high-resolution EEG and fMRI, this study is positioned to provide a multimodal characterization of how tACS influences both oscillatory activity and large-scale network interactions. EEG will allow for the assessment of immediate entrainment and aftereffects, including changes in spectral power, PLV, and ERPs. fMRI, in turn, will assess the spatial and functional reconfiguration of networks via seed-based connectivity and independent component analysis. Particular attention will be paid to connectivity within and between the DLPFC, ACC, insula, amygdala, and striatum. It is expected that alpha-band tACS applied to the DLPFC will enhance top-down control and strengthen connectivity with subcortical structures involved in rewards regulation, while theta-band stimulation of the ACC and insula may normalize aberrant activity in the impulsive and interoceptive systems. This convergence of oscillatory and connectivity-based evidence may validate tACS as a network-level intervention for food craving regulation.

Moreover, the integration of behavioral data, including validated craving questionnaires and Go/No-Go task performance, will allow for a direct correlation between neurophysiological changes and observable cognitive and affective outcomes. The inclusion of both trait- and state-level craving assessments (T-FCQ, S-FCQ, FCI) strengthens the design by capturing both enduring tendencies and momentary fluctuations in craving behavior. When combined with EEG and fMRI findings, these behavioral metrics will provide a comprehensive, multidimensional index of tACS efficacy.

Despite these strengths, several limitations warrant consideration. The relatively brief intervention duration of seven sessions may not fully capture the long-term potential of tACS to modify eating behaviors. Longitudinal studies with extended intervention periods are needed to examine the durability of neural and behavioral changes. Furthermore, individual variability in tACS responsiveness remains a challenge. Factors such as baseline oscillatory state, cortical anatomy, and skull conductivity can influence current distribution and entrainment efficacy (Saturnino et al., 2017). Although individualized modeling using structural MRI and the ROAST pipeline will optimize electrode placement and predict electric field strength, future research should explore real-time, closed-loop tACS protocols tailored to ongoing neural dynamics.

Another limitation relates to the inherent temporal and spatial trade-offs of EEG and fMRI. While EEG provides high temporal resolution, it cannot localize activity with the precision of fMRI. Conversely, fMRI offers excellent spatial resolution but limited insight into fast oscillatory dynamics. Simultaneous EEG-fMRI recordings could enhance future studies by capturing both the timing and spatial footprint of tACS-induced network changes. Moreover, functional connectivity measures are inherently correlational and cannot establish causality. Incorporating task-based fMRI with causal modeling approaches may provide a more mechanistic understanding of network reorganization.

A limitation of the within-subject design for the FA^–^ group is the absence of a washout period between active and sham sessions, which is also acknowledged. Although the sessions are counterbalanced and scheduled 24 h apart, previous research suggests that tACS aftereffects, especially at theta frequencies, may persist for several hours and potentially influence subsequent neural activity or behavioral responses (Herrmann et al., 2013; Wischnewski and Schutter, 2015). This temporal proximity may introduce residual effects, which will be accounted for in statistical analyses and acknowledged in interpretation.

It is also important to acknowledge that the study targets cortical regions involved in craving regulation. However, the broader aim is to engage large-scale neural networks that underlie self-control, salience detection, and interoceptive awareness. Although tACS cannot directly stimulate deep structures like the striatum or amygdala, frequency-specific entrainment of cortical hubs, such as the DLPFC, ACC, and insula, may drive network-level reorganization through their anatomical and functional connectivity. These regions are key nodes in the Salience Network, which governs the detection and prioritization of internal and external stimuli, and dynamically interacts with both the Default Mode Network and Executive Control Network (Parvaz et al., 2011; Volkow and Morales, 2015; Koob and Volkow, 2016).

The integration of EEG, fMRI, and behavioral data is expected to yield converging evidence on the neural mechanisms of tACS-induced craving modulation. We anticipate that frequency-specific changes in EEG spectral power and phase-locking will correlate with enhanced connectivity in prefrontal-subcortical networks observed via fMRI, and with improvements in behavioral indices of craving control, such as reduced subjective craving and improved inhibitory task performance. These multimodal relationships will help to validate neural markers that predict treatment responsiveness. Importantly, by using established craving scales and ecologically valid tasks, this protocol enhances the translational potential of tACS interventions in real-world settings, such as clinical programs targeting obesity or compulsive eating.

Finally, while this study focuses on food cravings, the theoretical framework and methodological approach have potential relevance to a broader range of compulsive and addictive behaviors. Given the shared neurocognitive mechanisms underlying substance use disorders, binge eating disorder, and impulse control deficits (Volkow and Morales, 2015; Volkow et al., 2019), findings from this protocol may support future clinical applications of tACS across multiple domains of addiction medicine.

In conclusion, this study protocol outlines a novel, multimodal investigation of the efficacy of tACS in modulating craving-related network activity, cognitive control, and subjective experience. By combining EEG, fMRI, and behavioral data within a rigorously controlled experimental design, it seeks to elucidate the neurophysiological mechanisms underlying craving regulation and the potential of oscillation-based neuromodulation for clinical intervention. If the anticipated effects are confirmed, this study will lay the groundwork for more personalized, frequency-targeted treatments for FA and related disorders, advancing both the theoretical understanding and therapeutic potential of non-invasive brain stimulation.

## 4 Ethics and dissemination

This study will be conducted in accordance with the ethical principles outlined in the Declaration of Helsinki and complies with international guidelines for human subject research. Ethical approval has been sought from both the Abu Dhabi Health Research and Technology Committee (ADHRTC) and the Human Research Ethics Committee of the United Arab Emirates University (UAEU-HREC). Final approval will be obtained prior to initiating participant enrollment.

All participants will provide written informed consent prior to participation. During the consent process, participants will be informed of the study’s objectives, procedures, potential risks and benefits, confidentiality safeguards, and their right to withdraw at any time without penalty or loss of entitlements. Consent will be obtained in a private setting and documented electronically and in writing.

To ensure participant safety, rigorous exclusion criteria have been implemented to minimize risks associated with tACS and MRI procedures. Adverse events will be closely monitored throughout the study, and any serious or unexpected effects will be reported to the ethics committees in accordance with regulatory standards. Participants experiencing adverse effects will be offered appropriate support and may be withdrawn from the study if necessary.

Confidentiality will be maintained by assigning a unique study ID to each participant. All identifiable data will be securely stored and accessible only to authorized members of the research team. De-identified data will be used for analysis, publication, and data sharing. Data handling and storage procedures will comply with institutional and national data protection regulations.

Any protocol amendments, including changes to eligibility, intervention procedures, or outcome assessments, will be submitted for ethics committee approval and documented in trial records. The study will be registered in a publicly accessible clinical trial registry before participant enrollment, and findings will be disseminated through peer-reviewed publications and conference presentations in accordance with open science principles.
